# Unlocking the potential of immunotherapy for patients with resectable non–small cell lung cancer

**DOI:** 10.3389/fonc.2025.1690367

**Published:** 2026-01-12

**Authors:** Sandip Pravin Patel, Justin Gainor, Steven Kao, Se-Hoon Lee, Tony Mok, Riyaz Shah, Caicun Zhou, Solange Peters

**Affiliations:** 1 UC San Diego Moores Cancer Center, San Diego, CA, United States; 2 Massachusetts General Hospital, Boston, MA, United States; 3 Chris O’Brien Lifehouse, Camperdown, NSW, Australia; 4 Samsung Medical Center, Sungkyunkwan University School of Medicine, Seoul, Republic of Korea; 5 State Key Laboratory of Translational Oncology, Department of Clinical Oncology, TheChinese University of Hong Kong, Hong Kong, Hong Kong SAR, China; 6 Maidstone Hospital, Maidstone, Kent, United Kingdom; 7 Shanghai East Hospital, Shanghai, China; 8 Lausanne University Hospital, Lausanne, Switzerland

**Keywords:** immunotherapy, resectable, NSCLC, locally advanced, neoadjuvant, perioperative, adjuvant, PD-L1

## Abstract

Following positive results in advanced and metastatic non–small cell lung cancer (NSCLC), there has been a move toward the application of immunotherapy in the treatment of locally advanced, resectable, oncogene driver–negative disease. To date, there have been eight Phase III trials across the adjuvant, neoadjuvant, and perioperative settings that demonstrate benefit with (chemo)-immunotherapy in patients with resectable NSCLC. Given the wealth of immunotherapy treatment regimens both available and under investigation in this setting, there is a need to determine the optimal timing of immunotherapy treatment (neoadjuvant, perioperative, or adjuvant) across disease stages to aid clinical decision-making. Established treatment guidelines often diverge, highlighting the need for a multidisciplinary team approach and consensus decision-making based on the latest evidence in the resectable setting. Finally, there is an unmet need surrounding the role of key predictive factors and response assessments, to assist clinicians in selecting patients for immunotherapy regimens. The aim of this review is to evaluate the current data and key considerations surrounding immunotherapy for the treatment of resectable NSCLC, including key parameters to inform de-escalating and escalating treatment approaches.

## Graphical Abstract

**Figure:**
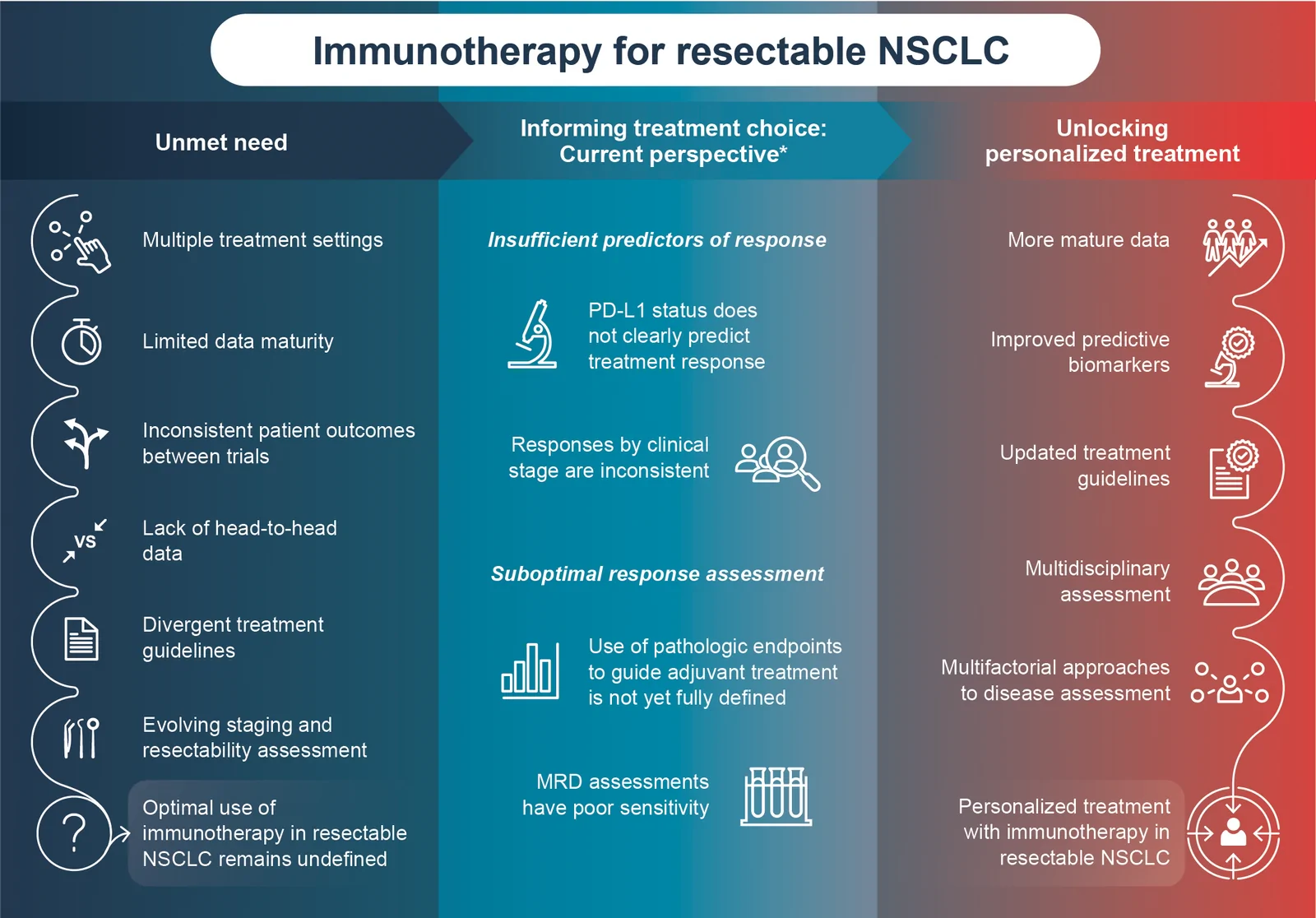
*In the absence of actionable *EGFR* and *ALK* alterations. MRD, minimal residual disease; NSCLC, non–small cell lung cancer; PD-L1, programmed death-ligand 1.

## 1 Introduction

Of the estimated 2.2 million people diagnosed with lung cancer each year, roughly 85% are diagnosed with non–small cell lung cancer (NSCLC); of these, 25%–30% are considered resectable (1–3). Surgery has traditionally been the standard of care for these patients; however, recurrence rates with surgery alone are high (30%–55%) (4–6). Patients with resected NSCLC also experience a higher risk of distant metastasis than local or regional recurrence, highlighting the need to target systemic control from the outset of treatment (7, 8). Compared with surgery alone, the addition of neoadjuvant or adjuvant chemotherapy is associated with only a modest improvement in 5-year survival (of approximately 4%–5%) in patients with resectable, locally advanced disease and can bring additional issues related to poor tolerability (8, 9). Therefore, there remains a need for more efficacious therapeutic options to improve survival in this population.

Following the success of immunotherapy in advanced and metastatic NSCLC, there has been a shift toward the application of immunotherapy (primarily, immune checkpoint inhibitors [ICIs] targeting programmed-death protein 1 [PD-1] or its ligand [PD-L1]) as first-line treatment for resectable disease without detectable *EGFR* or *ALK* mutations (10). A neoadjuvant regimen containing immunotherapy is thought to prime antitumorigenic activity to enhance systemic control and improve post-surgical outcomes (7). In the adjuvant setting, immunotherapy facilitates targeting of residual and/or micrometastatic disease following resection, leading to improvements in long-term disease control (7). However, determining the optimal approach to immunotherapy across disease stages, including the timing and duration of treatment, remains a challenge.

The aim of this review is to provide an overview of immunotherapy strategies in oncogene driver–negative, resectable NSCLC, including the current breadth of data available across adjuvant, neoadjuvant, and perioperative settings and the factors that can guide clinicians in selecting the optimal immunotherapy treatment approach.

## 2 Current immunotherapy guidelines for Stage II/III resectable NSCLC

**Figure 1:**
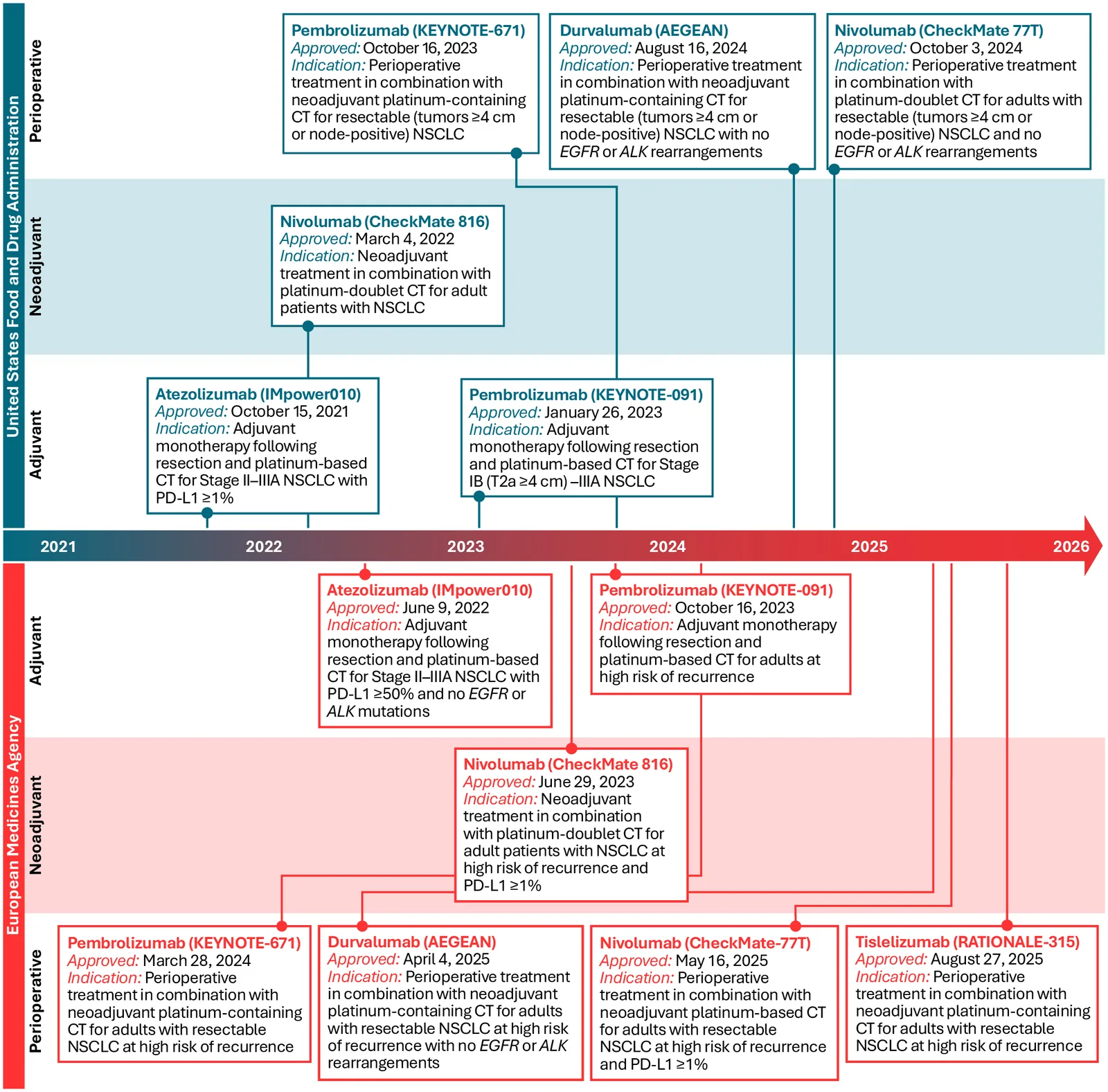
Timeline of key approvals of immunotherapy for the treatment of resectable NSCLC. CheckMate 816, IMpower010, and PEARLS/KEYNOTE-091 approvals followed enrollment based on the American Joint Committee on Cancer and the Union for International Cancer Control 7^th^ edition. AEGEAN, CheckMate 77T, and KEYNOTE-671 approvals followed enrollment based on the American Joint Committee on Cancer and the Union for International Cancer Control 8^th^ edition. CT, chemotherapy; NSCLC, non–small cell lung cancer; PD-L1, programmed-death ligand 1.

Neoadjuvant, adjuvant, and perioperative approval of immunotherapeutic agents for the treatment of resectable NSCLC are shown in **Figure 1**. The National Comprehensive Cancer Network (NCCN) and International Association for the Study of Lung Cancer (IASLC) updated their guidelines in 2024 and 2025, respectively, to reflect this changing landscape (both based on the American Joint Committee on Cancer [AJCC] and the Union for International Cancer Control [UICC] 8^th^ edition of lung cancer staging), and in 2024 the American Society of Clinical Oncology (ASCO) issued updated guidance on the treatment of Stage III disease (based on AJCC-UICC 7^th^ edition) (6, 7, 11, 12). To date, ASCO guidelines have not yet been updated to include perioperative immunotherapy (11, 12).

In all patients, testing for PD-L1 expression as well as for *EGFR* or *ALK* alterations is recommended prior to treatment initiation (6, 7, 11). Immunotherapy is not recommended for patients with actionable *EGFR* or *ALK* alterations, for which targeted adjuvant therapies are the standard of care. Only the NCCN guidelines refer to driver mutations other than *ALK* or *EGFR*, with targeted therapies remaining the recommended approach for these patients (6).

### 2.1 Stage III disease

For resectable Stage IIIA disease, recommendations for neoadjuvant chemoimmunotherapy are clear, with IASLC, ASCO, and NCCN guidelines recommending neoadjuvant chemoimmunotherapy for patients regardless of PD-L1 status. There is also broad agreement on the use of adjuvant chemoimmunotherapy in patients with PD-L1 ≥1% who did not receive neoadjuvant therapy; however, notably, the PEARLS/KEYNOTE-091 regimen is approved for an all-comer population (6, 7, 11). Where these guidelines differ is in their recommendation of perioperative chemoimmunotherapy. The NCCN guidelines recommend perioperative immunotherapy in accordance with the United States Food and Drug Administration (FDA) label (6). The IASLC guidelines do not discuss this approach in detail, stating that adjuvant immunotherapy can be considered following neoadjuvant chemoimmunotherapy, but to date these only cite inconsistent efficacy data in PD-L1 <1% and 1%–49% subgroups in adjuvant/perioperative trials (7).

### 2.2 Stage II disease

Recommendations for ICI treatment in resectable Stage II NSCLC diverge further. The NCCN guidelines on neo-/adjuvant ICI make no distinction between Stage II and Stage IIIA disease (6). However, the IASLC guidelines could not reach a consensus on neoadjuvant chemoimmunotherapy treatment of Stage II patients. Only 65% of the panel supported recommending neoadjuvant chemoimmunotherapy regardless of PD-L1 expression. Opponents cited a lack of sufficiently convincing or robust evidence supporting chemoimmunotherapy in these patients, as well as a risk that some patients may not then proceed to surgery (5, 7).

## 3 Resectability

The results of staging and resectability decisions directly inform the downstream management of the patient. Patients with resectable, oncogene driver–negative tumors are offered surgery with the option of adjuvant treatment, or neoadjuvant treatment followed by surgery and the potential for adjuvant therapy, as appropriate (7). By contrast, per the NCCN guidelines, chemoradiotherapy followed by consolidation immunotherapy represents the current standard of care for patients with Stage III unresectable tumors (6). As a result, accurate staging is essential to ensure that patients receive optimal treatment.

Preoperative, invasive staging of the hilar and mediastinal lymph nodes is commonly performed to assess the extent of spread to the intrapulmonary (N1) and mediastinal (N2 and N3) lymph nodes and is considered critical to inform optimal management for patients with potentially resectable NSCLC (13, 14). Minimally invasive techniques have become increasingly relied upon as accurate and cost-effective replacements for invasive staging procedures; however, there remain concerns over their sensitivity compared with traditional staging methodology (15). The latest guidelines on mediastinal staging from the European Society of Thoracic Surgeons recommend that if computed tomography (CT) or positron emission tomography results are suggestive of spread to mediastinal lymph nodes, tissue confirmation is required via minimally invasive techniques such as endosonography (endobronchial and/or esophageal ultrasonography) with fine needle aspiration or, in the case of negative results, invasive staging (16). Patients who are found to have extracapsular disease (visualized via mediastinoscopy) or bulky N2 disease (revealed by CT staging) are currently deemed to be unresectable (16).

Locally advanced NSCLC (Stages IIIA–IIIC) historically represents a challenging patient subset when determining resectability (17). Locally advanced disease may be classified as resectable, borderline resectable, or unresectable depending on local guidelines and the level of experience of the examining thoracic surgeon, potentially leading to discrepancies in the treatment that patients receive between surgeons, centers, and countries (17). Furthermore, the timing of assessment with respect to treatment is a critical factor influencing resectability determination.

In recent years, the use of neoadjuvant therapy to downstage borderline unresectable NSCLC has been explored to allow patients to access surgical resection (18). In particular, neoadjuvant chemoimmunotherapy has proven viable as conversion therapy in Stage IIIA–IIIB NSCLC (19). Although this represents an exciting option for patients with upfront unresectable disease, there remains a need to more accurately identify patients who would benefit and for further data on the validity of this approach. As a result, the use of neoadjuvant tumor downstaging is not currently recommended by the Society of Thoracic Surgeons, and neoadjuvant and perioperative immunotherapeutic approaches should be restricted to patients who are resectable at the time of initial workup (20).

Determination of tumor resectability and the operability of the patient is primarily the responsibility of the thoracic surgeon (20). However, given the increasing interplay between treatment and surgery, input from multiple specialties is crucial to decide optimal treatment options, including the feasibility and appropriateness of resection as well as the suitability of induction therapy or alternative treatment (20). As such, guidelines increasingly recommend the need for input from a multidisciplinary team spanning the lung cancer care pathway to accurately stratify patients and develop a personalized treatment plan (6, 7, 21).

## 4 Current evidence supporting immunotherapy treatment for resectable NSCLC

### 4.1 Adjuvant immunotherapy

Owing to high rates of recurrence after surgery in patients with locally advanced NSCLC, adjuvant systemic therapy is recommended for patients with resected Stage II–IIIA disease and may be considered for patients with Stage IB T2a tumors measuring 4 cm (AJCC-UICC 8^th^ edition) (6). Immunotherapy has been investigated in the adjuvant setting for resectable NSCLC, with clinically meaningful benefits in disease-free survival (DFS) identified in Phase III trials (**Table 1**) (22–25). The majority of trials investigate immunotherapy with sequential or concurrent chemotherapy in Stage IB–IIIA NSCLC and resectable Stage IIIB NSCLC (10). To date, the only Phase III trials that have read out positively in this setting are IMpower010 and PEARLS/KEYNOTE-091, which both enrolled patients per AJCC-UICC 7^th^ edition (22, 24). These trials demonstrated that adjuvant atezolizumab or pembrolizumab significantly improved DFS in patients with Stage IB–IIIA NSCLC following complete resection and platinum-based chemotherapy as compared with best supportive care (BSC) or placebo, respectively. In IMpower010, immature overall survival (OS) data did not indicate benefit in the intention-to-treat (ITT) population (stratified hazard ratio [HR]: 0.97 [95% confidence interval (CI): 0.78–1.22]) but were suggestive of benefit in patients with PD-L1 ≥50% Stage II–IIIA NSCLC (HR: 0.47 [95% CI: 0.28–0.77]) (23). In PEARLS/KEYNOTE-091, median OS was not reached in either group at the time of the interim analysis (HR: 0.87 [95% CI: 0.67–1.15]; *p* = 0.17) (24). Atezolizumab and pembrolizumab were subsequently approved for the adjuvant treatment of resectable NSCLC by both the FDA and the European Medicines Agency (EMA); however, the labels for atezolizumab differ in their restriction by PD-L1 expression level (tumor cell ≥1% for the FDA and ≥50% for the EMA), and the FDA label for pembrolizumab is restricted by tumor size (≥4 cm) (26–29). Notably, PEARLS/KEYNOTE-091 also revealed no DFS benefit in the subgroup of patients who did not receive adjuvant chemotherapy prior to treatment with adjuvant pembrolizumab (HR: 1.16 [95% CI: 0.73–1.84]) (7, 24, 30). As a result, guidelines from the IASLC and NCCN currently recommend that immunotherapy is received after adjuvant platinum-containing chemotherapy (6, 7).

**Table 1:** Key Phase III adjuvant immunotherapy trials in resectable NSCLC.

Trial	Treatment arms	mDFS (95% CI), mo	mOS (95% CI), mo	PD-L1, mDFS (95% CI), mo				Clinical stage, mDFS (95% CI), mo	
				<1%	1%–49%	≥50%	≥1%	II	III
IMpower010 (NCT02486718) (22, 23)	Adjuvant platinum-doublet chemotherapy followed by adjuvant atezolizumab ×16 cycles q3w (n=507)	NE (36.1–NE)	NR	36.1 (30.2–NE)	32.8 (29.4–NE)	NE (42.3–NE)	NE (36.1–NE)	IIA: NR (36.7–NR) IIB: 37.1 (32.3–NR)	IIIA: 32.3 (25.4–NE)
	Adjuvant platinum-doublet chemotherapy ×4 cycles q3w, followed by BSC (n=498)	35.3 (29.0–NE)	NR	37.0 (28.6–NE)	31.4 (24.0–NE)	35.7 (29.7–NE)	35.8 (29.0–NE)	IIA: NE (31.0–NR) IIB: 46.4 (32.0–NR)	IIIA: 29.7 (23.7–35.3)
	HR (95% CI)	0.66 (0.50–0.88) *p* = 0.0039	0.97* (0.78–1.22)	0.97 (0.72–1.31)	0.87 (0.60–1.26)	0.43 (0.27–0.68)	0.65 (0.42–1.01)	IIA: 0.68 (0.46–1.00) IIB: 0.88 (0.54–1.42)	IIIA: 0.81 (0.61–1.06)
PEARLS/KEYNOTE-091 (NCT02504372) (24)	Adjuvant pembrolizumab q3w for 1 year with or without prior adjuvant platinum-doublet chemotherapy (n=590)	53.6 (39.2–NR)	–	–	–	–	–	–	–
	Adjuvant placebo q3w for 1 year with or without prior adjuvant platinum-doublet chemotherapy (n=587)	42.0 (31.3–NR)	–	–	–	–	–	–	–
	HR (95% CI)	0.76 (0.63–0.91) *p* = 0.0014	0.87* (0.67–1.15) *p* = 0.17	0.78 (0.58–1.03)	0.67 (0.48–0.92)	0.82 (0.57–1.18)	–	0.70 (0.55–0.91)	0.92 (0.69–1.24)
ADJUVANT BR.31 (NCT02273375) (25)	Optional adjuvant chemotherapy followed by adjuvant durvalumab ×12 cycles q4w^†^	60 (50–78)	NR	NR	NR	NR	60 (48–78)	NR	NR
	Optional adjuvant chemotherapy followed by adjuvant placebo ×12 cycles q4w^†^	54 (37–67)	NR	NR	NR	NR	60 (44–81)	NR	NR
	HR (95% CI)	0.89 (0.75–1.07) *p* = 0.21^‡^	–	–	–	–	0.99 (0.79–1.25) *p* = 0.93^‡^	–	–

IMpower010 and PEARLS/KEYNOTE-091 enrolled patients based on the American Joint Committee on Cancer and the Union for International Cancer Control 7^th^ edition.

*Based on immature OS data. ^†^Data are presented for patients with no *EGFR* or *ALK* aberrations only (n=1219; total enrolled population, n=1415). ^‡^Primary endpoint was DFS in PD-L1 ≥25%, mDFS (95% CI): durvalumab, 70 (58–NR) mo, and placebo, 60 (48–NR) mo; HR: 0.94 (95% CI: 0.71–1.25); *p* = 0.64.

BSC, best supportive care; CI, confidence interval; DFS, disease-free survival; HR, hazard ratio; mDFS, median disease-free survival; mo, months; mOS, median overall survival; NE, not evaluable; NR, not reported; OS, overall survival; PD-L1, programmed-death ligand 1; q3w, every 3 weeks; q4w, every 4 weeks.

The only other Phase III trial that has read out in this setting is the ADJUVANT BR.31 trial of durvalumab versus placebo in patients with Stage IB–IIIA NSCLC (AJCC-UICC 7^th^ edition) who had received surgery with or without adjuvant platinum-based chemotherapy. ADJUVANT BR.31 failed to meet the primary endpoint of improved DFS with adjuvant durvalumab in patients with PD-L1 ≥25%, and it remains to be determined if the demanding surgical requirements influenced this negative result (31). An additional two trials investigating adjuvant durvalumab in resectable disease, MERMAID-1 and MERMAID-2, were terminated shortly after initiation owing to multiple approvals in the perioperative setting and barriers in implementing sensitive and practically manageable tumor-informed minimal residual disease (MRD) measurements, which resulted in inconclusive efficacy data because of the small sample size and limited follow-up (32–34). Further trials (including the ANVIL trial of nivolumab following adjuvant chemotherapy in Stage IB–IIIA NSCLC (AJCC-UICC 7^th^ edition), the ALCHEMIST trial of concurrent adjuvant pembrolizumab plus platinum-based chemotherapy in Stage IIA–IIIB NSCLC (AJCC-UICC 8^th^ edition), the open-label NADIM-ADJUVANT trial of adjuvant nivolumab plus chemotherapy followed by maintenance nivolumab in Stage IB–IIIA NSCLC (AJCC-UICC 8^th^ edition), and the LungMate-008 trial of adjuvant toripalimab plus chemotherapy in Stage II–IIIB NSCLC (AJCC-UICC 8^th^ edition), will continue to inform on the feasibility of adjuvant immunotherapy in this setting (35–38).

### 4.2 Neoadjuvant immunotherapy

There is a strong rationale supporting the evaluation of ICIs in the neoadjuvant setting, as immunotherapy is known to prime the immune system to target tumor cells (39). Early induction of an antitumorigenic response in the neoadjuvant setting could therefore facilitate timely and sustained targeting of micrometastatic disease, reducing the risk of metastasis following surgery (39). Furthermore, it is thought that an increased immunological response and enhanced immunogenic cell death takes place when the localized tumor is intact, as this represents a large antigen burden and facilitates more comprehensive T-cell activation (40–42). Taken together, the earlier and stronger immune response with neoadjuvant therapy facilitates more effective suppression of metastasis and helps to prevent recurrence following surgical resection (39, 42).

Neoadjuvant immunotherapy for resectable NSCLC was first investigated in a single-arm pilot study by Forde PM, et al. in 2018 (39). In this study, two cycles of neoadjuvant nivolumab followed by surgery resulted in a major pathologic response (MPR) rate of 45% compared with a historical 15% MPR rate with neoadjuvant chemotherapy (39). Following this pilot, a number of Phase II trials began to investigate immunotherapy as monotherapy and in combination with other agents (7).

In particular, impressive results have been found when evaluating neoadjuvant chemoimmunotherapy for resectable NSCLC; these are summarized in **Table 2**. Notably, CheckMate 816 was the first Phase III trial to evaluate neoadjuvant chemoimmunotherapy in this setting, investigating three cycles of neoadjuvant nivolumab plus platinum-doublet chemotherapy in patients with Stage IB–IIIA NSCLC (AJCC-UICC 7^th^ edition) (5). At the first pre-specified analysis, the study found an event-free survival (EFS) benefit for the combination regimen compared with neoadjuvant chemotherapy alone following a minimum of 21 months of follow-up (HR: 0.63 [97.38% CI: 0.43–0.91]; *p =*0.005) (5). The study yielded impressive landmark EFS results, with 2-year EFS rates of 63.8% versus 45.3% and 3-year EFS rates of 57% versus 43% in the treatment versus control arms, respectively (45). In addition, the rate of pathologic complete response (pCR) was significantly higher with neoadjuvant chemoimmunotherapy than with chemotherapy alone (odds ratio: 13.94 [99% CI: 3.49–55.75]; *p*< 0.001) (5). At the latest (4-year) update, EFS benefits of added nivolumab persisted (4-year EFS rates: 49% for neoadjuvant nivolumab plus chemotherapy versus 38% for neoadjuvant chemotherapy; HR for EFS: 0.66 [95% CI: 0.49–0.90]) (43). At the pre-planned, final, 5-year analysis of CheckMate 816, a statistically significant and clinically meaningful OS benefit was demonstrated in resectable NSCLC (HR: 0.72 [95% CI: 0.523–0.998]; *p* = 0.048) (44). At this timepoint, median OS had not yet been reached in the nivolumab arm, compared with 73.7 months (95% CI: 47.3–NR) in the chemotherapy arm. Nivolumab represents the only immunotherapy agent approved for neoadjuvant use in resectable NSCLC in combination with platinum-doublet chemotherapy; although it is approved for all patients with either node-positive disease or tumors ≥4 cm in the United States, the EMA label is restricted to PD-L1 ≥1% in this setting (46, 47).

**Table 2:** Key Phase III neoadjuvant immunotherapy trial in resectable NSCLC.

Trial	Treatment arms	pCR, %	MPR, %	mEFS (95% CI), mo	mOS (95% CI), mo	PD-L1, mEFS (95% CI), mo				Clinical stage, mEFS (95% CI), mo	
						<1%	1%–49%	≥50%	≥1%	II	III
CheckMate 816 (NCT02998528) (5, 43, 44)	Neoadjuvant nivolumab + platinum-doublet chemotherapy ×3 cycles q3w (n=179)	–	–	43.8 (30.6–NR)	NR	25.1 (14.6–NR)	NR (27.8–NR)	NR (NR–NR)	NR (NR–NR)	NR (27.8–NR)	IIIA: 31.6 (26.6–NR)
	Neoadjuvant platinum-doublet chemotherapy ×3 cycles q3w (n=179)	–	–	18.4 (14.0–26.7)	73.7 (47.3–NR)	18.4 (13.9–26.2)	26.7 (11.5–NR)	19.6 (8.2–NR)	21.1 (11.5–NR)	NR (16.8–NR)	IIIA: 15.7 (10.8–22.7)
	HR (95% CI)	–	–	0.66 (0.49–0.90)	0.72 (95% CI: 0.51–0.98) *p* = 0.048	0.85 (0.54–1.32)	0.58 (0.30–1.12)	0.24 (0.10–0.61)	0.41 (0.24–0.70)	0.87 (0.48–1.56)	IIIA: 0.54 (0.37–0.80)

CheckMate 816 enrolled patients based on the American Joint Committee on Cancer and the Union for International Cancer Control 7^th^ edition.

CI, confidence interval; EFS, event-free survival; HR, hazard ratio; mEFS, median event-free survival; mo, months; mOS, median overall survival; MPR, major pathologic response; NR, not reported; OS, overall survival; pCR, pathologic complete response; PD-L1, programmed-death ligand 1; q3w, every 3 weeks.

Although the advent of immunotherapy-based neoadjuvant treatment has increased the number of patients with improved outcomes following surgery, it is associated with several challenges. Primarily, these center around the risk that patients may be unable to proceed with planned surgery owing to treatment-related toxicities or disease progression that occurs while they are receiving the neoadjuvant treatment, or withdrawal of patient consent (48). Across trials that include neoadjuvant chemoimmunotherapy, up to 20% of patients do not proceed to surgery owing to disease progression, treatment-related toxicity, patient or investigator decision, or later classification of tumors as inoperable. In addition, the complexity of surgery may be increased following receipt of neoadjuvant immunotherapy, owing to the potential for inflamed lymph nodes, hilar fibrosis, and increased frailty of pulmonary and vascular structures (49, 50). Rates of surgical cancellations, surgical delays, and conversion rates from minimally invasive to open procedures vary in the published literature; however, overall, neoadjuvant immunotherapy is not considered to substantially affect surgical outcomes in suitably selected patients (51). Finally, there is also a need to define the role of radiotherapy for patients eligible for a neoadjuvant immunotherapy–containing regimen. Phase II trial data have indicated that sub-ablative stereotactic radiotherapy may enhance the immune response to neoadjuvant immunotherapy (52). Future studies in a larger patient population will be important to inform on the role of radiotherapy in combination with immunotherapy.

### 4.3 Perioperative immunotherapy

A perioperative regimen makes use of immunotherapy in both the neoadjuvant and the adjuvant phases, to ensure continued targeting of micrometastatic disease with the intent of reducing the incidence of post-surgical recurrence (53). Several Phase III trials have been conducted to date (**Table 3**) and have revealed positive results; based on these, both the NCCN and the IASLC recommend that patients with Stage IB–IIIA NSCLC (AJCC-UICC 8^th^ edition) be evaluated for perioperative immunotherapy (6, 7).

**Table 3:** Key Phase III perioperative immunotherapy trials in resectable NSCLC.

Trial	Treatment arms	pCR, %	MPR, %	mEFS (95% CI), mo	mOS (95% CI), mo	PD-L1, mEFS (95% CI), mo				Clinical stage, mEFS (95% CI), mo	
						<1%	1%–49%	≥50%	≥1%	II	III
KEYNOTE-671 (NCT03425643) (54, 55)	Neoadjuvant pembrolizumab + platinum-doublet chemotherapy ×4 cycles q3w, followed by adjuvant pembrolizumab ×13 cycles q3w (n=397)	–	–	57.1 (38.0–69.1)	NR (NR–NR)	–	–	–	–	–	–
	Neoadjuvant placebo + platinum-doublet chemotherapy ×4 cycles q3w, followed by adjuvant placebo ×13 cycles q3w (n=400)	–	–	18.4 (14.8–22.1)	NR (53.0–NR)	–	–	–	–	–	–
	HR (95% CI)	–	–	0.57 (0.47–0.69)	0.73* (0.58–0.92)	0.75 (0.56–1.01)	0.52 (0.36–0.73)	0.48 (0.33–0.71)	0.51 (0.39–0.66)	0.59 (0.40–0.88)	0.58 (0.46–0.72)
AEGEAN (NCT03800134) (56)	Neoadjuvant durvalumab + platinum-doublet chemotherapy ×4 cycles q3w, followed by adjuvant durvalumab ×12 cycles q4w (n=366)	17.2	–	NR (31.9–NR)	–	NR (14.9–NR)	NR (31.9–NR)	NR (NR–NR)	–	NR (NR–NR)	IIIA: NR (NR–NR) IIIB: 31.9 (11.7–NR)
	Neoadjuvant placebo + platinum-doublet chemotherapy ×4 cycles q3w, followed by adjuvant placebo ×12 cycles q4w (n=374)	4.3	–	25.9 (18.9–NR)	–	20.6 (13.9–NR)	25.4 (12.2–NR)	26.2 (14.3–NR)	–	31.1 (25.4–NR)	IIIA: 19.5 (11.7–NR) IIIB: 18.9 (11.8–NR)
	HR (95% CI)	Difference: 13.0 (8.7–17.6) *p*<0.001	–	0.68 (0.53–0.88) *p=*0.004	–	0.76 (0.49–1.17)	0.70 (0.46–1.05)	0.60 (0.35–1.01)	–	0.76 (0.43–1.34)	IIIA: 0.57 (0.39–0.83) IIIB: 0.83 (0.52–1.32)
NEOTORCH (NCT04158440) (57)	Neoadjuvant toripalimab + platinum-doublet chemotherapy ×3 cycles q3w, followed by adjuvant toripalimab + platinum-doublet chemotherapy ×1 cycle, followed by adjuvant toripalimab ×13 cycles q3w (Stage III, n=202)	–	48.5 (41.4–55.6)	NR (24.4–NR)	NE (NE–NE)	–	–	–	–	–	–
	Neoadjuvant placebo + platinum-doublet chemotherapy ×3 cycles q3w, followed by placebo + platinum-based chemotherapy ×1 cycle followed by adjuvant placebo ×13 cycles q3w (Stage III, n=202)	–	8.4 (5.0–13.1)	15.1 (10.6–21.9)	30.4 (29.2–NE)	–	–	–	–	–	–
	HR (95% CI)	–	Difference: 13.0 (8.7–17.6) *p*<0.001	0.40 (0.28–0.57) *p*<0.0001	0.62* (0.38–1.00)	–	–	–	–	–	–
CheckMate 77T (NCT04025879) (58, 59)	Neoadjuvant nivolumab + platinum-doublet chemotherapy ×4 cycles q3w, followed by adjuvant nivolumab ×12 cycles q4w (n=229)	–	35.4 (29.2–41.9)	46.6	–	40.1	40.1	NR	46.6	NR	42.1
	Neoadjuvant placebo + platinum-doublet chemotherapy ×4 cycles q3w, followed by adjuvant placebo ×12 cycles q4w (n=232)	–	12.1 (8.2–17.0)	16.9	–	19.8	18.4	8.0	15.1	NR	13.4
	HR (95% CI)	–	Unweighted difference: 23.3 (15.7–30.6)	0.61 (0.46–0.80)	–	0.79 (0.52–1.21)	0.74 (0.47–1.17)	0.30 (0.15–0.59)	0.53 (0.36–0.76)	0.77 (0.46–1.30)	0.54 (0.39–0.74)
RATIONALE-315 (NCT04379635) (60, 61)	Neoadjuvant tislelizumab + platinum-doublet chemotherapy ×3–4 cycles q3w, followed by adjuvant tislelizumab ×8 cycles q6w (n=226)	41 (34–47)	56 (50–63)	NR (NR–NR)	NR (NE–NE)	–	–	–	–	NR (NE–NE)	NR (29.6–NE)
	Neoadjuvant placebo + platinum-doublet chemotherapy ×3–4 cycles q3w, followed by adjuvant placebo ×8 cycles q6w (n=227)	6 (3–10)	15 (11–20)	NR (16.6–NR)	NR (NE–NE)	–	–	–	–	NR (16.6–NE)	19.8 (13.1–NE)
	HR (95% CI)	Difference: 35 (28–42) *p* < 0.0001	Difference: 41 (33–49) *p*< 0.0001	0.56 (0.40–0.79) *p* = 0.0003	0.65 (0.45–0.95) *p*= 0.009	0.80 (0.47–1.38)	0.34 (0.17–0.66)	0.71 (0.38–1.34)	0.50 (0.32–0.78)	0.47 (0.26–0.87)	IIIA: 0.62 (0.42–0.94)
IMpower030 (NCT03456063) [mentioned in (7)]	Neoadjuvant atezolizumab + platinum-doublet chemotherapy ×4 cycles q3w, followed by adjuvant atezolizumab ×16 cycles q3w	–	–	–	–	–	–	–	–	–	–
	Neoadjuvant placebo + platinum-doublet chemotherapy ×4 cycles q3w, followed by BSC	–	–	–	–	–	–	–	–	–	–
	HR (95% CI)	–	–	–	–	–	–	–	–	–	–

All trials enrolled patients based on the American Joint Committee on Cancer and the Union for International Cancer Control 8^th^ edition.

*Based on immature OS data.

BSC, best supportive care; CI, confidence interval; EFS, event-free survival; HR, hazard ratio; mEFS, median event-free survival; mo, months; mOS, median overall survival; MPR, major pathologic response; NE, not evaluable; NR, not reported; OS, overall survival; pCR, pathologic complete response; PD-L1, programmed-death ligand 1; q3w, every 3 weeks; q4w, every 4 weeks; q6w, every 6 weeks.

All perioperative immunotherapy trials summarized in **Table 3** have demonstrated an EFS benefit of immunotherapy versus comparator. Of these, pembrolizumab was the first immunotherapy approved in the perioperative setting in both Europe and the United States, based on the KEYNOTE-671 trial of perioperative pembrolizumab with neoadjuvant platinum-doublet chemotherapy compared with perioperative placebo plus neoadjuvant chemotherapy in patients with Stage II, IIIA, or IIIB NSCLC (AJCC-UICC 8^th^ edition) (62). Dual primary endpoints in KEYNOTE-671 were EFS and OS. In the planned interim analysis, the EFS endpoint was met, with a median EFS of 47.2 months (95% CI: 32.9 – not reported [NR]) with pembrolizumab versus 18.3 months (95% CI: 14.8–22.1) with placebo (HR: 0.59 [95% CI: 0.48–0.72]; *p*< 0.0001) (62). After a median follow-up of 3 years, KEYNOTE-671 became the first perioperative trial to demonstrate significant OS improvement (HR: 0.72 [95% CI: 0.56–0.93]; one-sided *p* = 0.0052 [one-sided threshold *p* = 0.0054]) (7, 54). At approximately 4 years of follow-up, the 48-month OS rate was 68.0% (95% CI: 63.0%–72.5%) with perioperative pembrolizumab versus 56.7% (95% CI: 51.2%–61.8%) with chemotherapy alone, and median OS had not been reached in either treatment arm (55). Most recently, tislelizumab was approved for use in the perioperative setting in Europe, based on the results of the RATIONALE-315 trial of neoadjuvant chemotherapy with either perioperative tislelizumab or perioperative placebo (63, 64). At interim analysis, RATIONALE-315 met its primary endpoints of improved MPR (between-group difference: 41% [95% CI: 33%–49%]; *p* < 0.0001) and improved EFS (HR: 0.56 [95% CI: 0.40–0.79]; *p* = 0.0003), alongside higher pCR (between-group difference: 35% [95% CI: 28%–42%]; *p* < 0.0001) (60). At final analysis, RATIONALE-315 became the second trial of perioperative immunotherapy to demonstrate statistically significant OS benefit in patients with resectable NSCLC (HR: 0.65 [95% CI: 0.45–0.93]; p = 0.009) (54, 61).

While significant OS results have not yet been reported, FDA and EMA approvals have been received for perioperative durvalumab and nivolumab based on the Phase III AEGEAN and CheckMate 77T trials, respectively (46, 47, 65, 66). Both trials met their primary endpoint of improved EFS versus chemotherapy alone, with an HR of 0.68 (95% CI: 0.53–0.88; *p* = 0.004) for durvalumab and 0.58 (97.36% CI: 0.42–0.81; *p* < 0.001) for nivolumab (56, 58). Furthermore, the NEOTORCH trial of neoadjuvant chemotherapy with either perioperative toripalimab or perioperative placebo showed improved EFS (HR: 0.40 [95% CI: 0.28–0.57]; *p* < 0.0001) and MPR (between-group difference: 13.0% [95% CI: 8.7%–17.6%]; *p* < 0.001) (57).

Although some of the clinical challenges associated with perioperative immunotherapy are similar to those that apply in the neoadjuvant setting, there are additional considerations influencing the use of perioperative therapy. Crucially, it remains to be determined how best to select patients who will benefit most from adjuvant immunotherapy following an initial neoadjuvant course of chemoimmunotherapy and thus avoid overtreatment (see *Selecting between treatment approaches in resectable NSCLC*) (48). Although retrospective analyses have found that patients who achieve pCR following neoadjuvant therapy may not experience additional benefit from further treatment in the adjuvant setting, this has not yet been tested prospectively (see *Response assessment in NSCLC and the potential for de-escalation/escalation of treatment*) (67). In addition, there is a need to define the optimal duration of neoadjuvant treatment in a perioperative regimen (48). To date, Phase III trials have primarily investigated three cycles (CheckMate 816) or four cycles (AEGEAN, KEYNOTE-671, and CheckMate 77T) of neoadjuvant chemoimmunotherapy (39, 56, 58). NEOTORCH investigated three cycles of neoadjuvant chemoimmunotherapy, followed by one cycle of adjuvant chemoimmunotherapy prior to adjuvant immunotherapy (57). Finally, RATIONALE-315 investigated between three and four cycles of chemoimmunotherapy in the neoadjuvant setting (60). This question will be examined prospectively in the Phase III neoSCORE II trial, which will explore three versus four cycles of neoadjuvant chemoimmunotherapy in patients with resectable squamous NSCLC, followed by adjuvant immunotherapy (48). Finally, the optimal duration of the adjuvant course of immunotherapy remains to be defined, although the majority of regimens complete postoperative immunotherapy at 1 year (48). As trials investigating perioperative immunotherapy mature, these data will be important to inform on these current challenges.

## 5 Key predictors of response

### 5.1 PD-L1 expression

PD-L1 is currently the most established and widely used predictive biomarker for immunotherapy in advanced NSCLC, and many trials in resectable NSCLC stratify patients based on PD-L1 expression status (68). Measurement of PD-L1 expression is recommended by the NCCN, ASCO, and IASLC guidelines prior to initiating treatment, along with *EGFR* and *ALK* mutational status (6, 7, 11). Although widely used, PD-L1 has been described as an imperfect biomarker because of its heterogeneous and dynamic expression and the varying cut-off values used to define PD-L1 positivity (68). Indeed, the prognostic value of PD-L1 expression for chemoimmunotherapy outcomes is not yet established, in part because of apparently conflicting evidence of immunotherapy benefit in patients with PD-L1 expression <1%, particularly in the adjuvant setting (5, 22, 62, 69).

IMpower010 and KEYNOTE-091 present a contrasting picture of the benefit of adjuvant immunotherapy across PD-L1 expression subgroups. In IMpower010, final DFS data revealed benefit with adjuvant atezolizumab versus BSC in patients with Stage II–IIIA NSCLC (AJCC-UICC 7^th^ edition) and PD-L1 ≥1%, which appeared to be driven by a benefit in the PD-L1 ≥50% population (**Table 1**) (22, 25). While not formally statistically tested, immature OS data were not suggestive of benefit in the ITT population (HR: 0.97 [95% CI: 0.78–1.22]) but trended towards benefit in the PD-L1 ≥50% subpopulation (HR: 0.43 [95% CI: 0.27–0.68]) (23). In contrast, in the KEYNOTE-091 trial, DFS was significantly improved in patients receiving pembrolizumab in the all-comer population, regardless of PD-L1 expression, but patients with PD-L1 expression ≥50% did not appear to exhibit a survival benefit owing to an unexpected overperformance of the control arm in this subgroup (**Table 1**) (24). Based on the sum of this evidence, guidelines from ASCO, the NCCN, and the IASLC recommend adjuvant immunotherapy for patients with PD-L1 expression ≥50% in tumor cells (6, 7, 11).

In the neoadjuvant setting, several neoadjuvant-only and perioperative studies indicate greater EFS/pCR outcomes with higher PD-L1 expression (**Tables 2**, **3**) (5, 57, 62). Indeed, based on the data from CheckMate 816, the EMA approved nivolumab in combination with platinum-based chemotherapy for patients with resectable NSCLC and tumor cell PD-L1 ≥1% (**Table 2**) (47). This restriction is not included in the FDA indication, and ex-European treatment guidelines recommend neoadjuvant immunochemotherapy regardless of PD-L1 expression (6, 7, 11, 46). Meta-analyses of studies evaluating neoadjuvant chemoimmunotherapy support the potential prognostic value of PD-L1 expression (70, 71). Mo D-C, et al. demonstrated that neoadjuvant chemoimmunotherapy was associated with improved pCR and EFS regardless of PD-L1 expression, compared with neoadjuvant chemotherapy; however, patients with higher PD-L1 expression may have a lower risk of disease progression than those with lower PD-L1 expression (71). Similarly, Banna GL, et al. demonstrated improved rates of pCR and 2-year EFS regardless of PD-L1 expression with neoadjuvant chemoimmunotherapy compared with neoadjuvant chemotherapy alone, as well as an apparent trend that higher PD-L1 expression was associated with increased rates of 2-year EFS compared with lower PD-L1 expression (70). The prognostic role of PD-L1 expression on OS is difficult to determine given the immaturity of the relevant data; however, there are indicators that improved OS following neoadjuvant chemoimmunotherapy is restricted to patients with PD-L1-positive tumors (71). Based on subgroup analyses of CheckMate 816, OS benefit with nivolumab plus chemotherapy appeared to be greatest in patients with PD-L1 expression ≥1% (HR: 0.51 [95% CI: 0.31–0.84]), although patients with PD-L1-negative tumors can still achieve pCR and durable remission while receiving this treatment regimen (5, 44).

Many studies of perioperative immunotherapy have also shown EFS improvement in patients with tumor cell PD-L1 ≥1% (**Table 3**) (54, 56, 58, 60). For AEGEAN and CheckMate 77T, EFS benefit appears to be driven by the PD-L1 ≥50% population (56, 58, 59). Furthermore, subgroup analysis of OS in KEYNOTE-671 indicated greatest benefit with perioperative pembrolizumab versus chemotherapy in patients with PD-L1 ≥50% (HR: 0.55 [95% CI: 0.33–0.92]) (54). Pembrolizumab was approved as a perioperative regimen in resectable NSCLC for an all-comer population by both the FDA and the EMA (26, 27, 54). By contrast, while the FDA approved perioperative nivolumab without PD-L1 restriction, the Committee for Medicinal Products for Human Use (CHMP) recently adopted a positive opinion for perioperative nivolumab for patients with PD-L1 ≥1% (46, 72). More mature data across perioperative trials will be important to inform the use of PD-L1 as a biomarker for patient selection.

As chemoimmunotherapy trials in resectable NSCLC further mature, it is likely that so too will the value of PD-L1 expression as a prognostic tool, further reinforcing the need to assess PD-L1 expression prior to treatment initiation. However, given the treatment benefit of chemoimmunotherapy observed in some PD-L1-negative patients, it will be important to identify additional predictive markers in patients with *EGFR* and *ALK* wild-type resectable NSCLC.

### 5.2 Clinical stage

Clinical stage has emerged as a potential prognostic factor for chemoimmunotherapy in resectable *EGFR* and *ALK* wild-type NSCLC. Stage IIIA NSCLC remains the clinical stage with the most consistent data, with EFS/DFS benefit in favor of neoadjuvant/perioperative chemoimmunotherapy demonstrated across Phase III trials (**Tables 2** and **3**) (5, 54, 56, 58, 60). Although Phase III adjuvant (chemo)-immunotherapy trials have reported significant DFS benefit versus chemotherapy (IMpower010 and KEYNOTE-091), neither have established clear benefit in patients with Stage III disease to date (**Table 1**) (22, 24). Currently, there is limited evidence for the survival benefit of immunotherapy in patients with Stage IIIB disease; no data have been reported for this subgroup to date in either the adjuvant or the neoadjuvant setting (**Tables 1**, **2**). Data from perioperative trials remain conflicted in Stage IIIB NSCLC (AJCC-UICC 8^th^ edition). No clear EFS benefit was observed for this subgroup in AEGEAN (HR: 0.83 [95% CI: 0.52–1.32]); however, EFS benefit for patients with Stage IIIB NSCLC was observed in NEOTORCH (HR: 0.30 [95% CI: 0.15–0.56]; *p <*0.001) and KEYNOTE-671 (HR: 0.57 [95% CI: 0.36–0.90]) (7, 56, 57). Sub-stage analyses of Stage IIIB NSCLC have not yet been reported for CheckMate 77T and will be important to provide further evidence on the utility of a perioperative approach in these patients.

Although it is clear that immunotherapy regimens have the potential to benefit patients with resectable Stage II NSCLC (AJCC-UICC 8^th^ edition), further data are required to inform on optimal treatment strategy. Subgroup analyses of the CheckMate 816 trial provided inconclusive evidence of a treatment benefit for the addition of nivolumab to neoadjuvant chemotherapy in patients with Stage IB or II NSCLC (AJCC-UICC 7^th^ edition; these would all be classified as Stage II per the AJCC-UICC 8^th^ edition) (5, 44). Although there was no clear EFS or OS benefit demonstrated (EFS HR: 0.87 [95% CI: 0.48–1.56]; OS HR: 0.77 [95% CI: 0.44–1.35]), pCR benefit was observed in this subpopulation (difference: 21.4% [95% CI: 9.0%–33.6%]). Recent meta-analyses suggest that neoadjuvant chemoimmunotherapy may result in a longer EFS in patients with Stage IIIA NSCLC than in those with Stage II NSCLC (70, 73). Despite this, a meta-analysis of eight randomized controlled trials suggested that neoadjuvant chemoimmunotherapy improved 2-year EFS compared with chemotherapy alone in Stage II NSCLC (HR: 0.75 [95% CI: 0.57–0.97]) (70). These data, along with the lack of sub-analyses by PD-L1 expression within Stage II disease, have resulted in some uncertainty in the best approach to treat Stage II patients (AJCC-UICC 8^th^ edition), as discussed in the IASLC treatment guidelines (7). In the perioperative setting, KEYNOTE-671 (HR: 0.59 [95% CI: 0.40–0.88]) and RATIONALE-315 (HR: 0.47 [95% CI: 0.26–0.87]) represent the only Phase III trials to demonstrate a clear EFS benefit in patients with Stage II NSCLC (54, 60). This benefit appeared to be mirrored by the Stage II OS findings in both trials (KEYNOTE-671 HR: 0.67 [95% CI: 0.41–1.10]; RATIONALE-315 HR: 0.58 [95% CI: 0.31–1.06]) (7, 61). Finally, in the adjuvant setting, KEYNOTE-091 found DFS benefit with pembrolizumab versus chemotherapy in patients with Stage II NSCLC (HR: 0.70 [95% CI: 0.55–0.91]), whereas IMpower010 found that DFS benefit with atezolizumab versus chemotherapy in Stage II NSCLC appeared to be restricted patients with Stage IIA, rather than Stage IIB, disease (**Table 1**) (22–24).

Stage III N2 disease represents a subgroup of particular clinical interest owing to its potentially curable nature, although prognosis is historically poor and optimal treatment remains undefined (74). In an exploratory analysis of CheckMate 77T, patients with Stage III NSCLC appeared to experience EFS benefit regardless of nodal status, with particular benefit in patients with N2 NSCLC (HR: 0.48 [95% CI: 0.32–0.72]) (59). Immature data showed that patients with and without N2 disease showed higher rates of pCR following treatment with perioperative nivolumab versus chemotherapy (22.0% versus 5.6%, and 25.5% versus 5.3%, respectively) (75). Surgical feasibility also appeared to be similar between patients with and without N2 disease, and over half of patients with Stage III NSCLC experienced nodal downstaging with perioperative nivolumab (75). In AEGEAN, exploratory analysis revealed that patients with N2 disease showed EFS benefit with perioperative durvalumab versus chemotherapy (HR: 0.63 [95% CI: 0.43–0.90]), with higher rates of pCR (16.6% versus 4.9%, respectively) and MPR (32.6% versus 15.1%, respectively) (76). The presence of N2 disease did not appear to have a significant impact on delays to surgery or the type of surgery received (76).

Finally, of clinical interest is the treatment of Stage III single-station versus multi-station N2 NSCLC. Patients undergoing surgical resection of Stage III single-station N2 NSCLC were found to have a higher rate of 5-year OS relative to those with multi-station N2 NSCLC (~35% versus 20%, respectively), leading some to propose that single- versus multi-station disease may be useful as a prognostic factor for survival outcomes (77). In the Phase II NADIM II trial, approximately two-thirds of the patients had pathologically confirmed N2 disease and 38.4% had multi-station N2 (78). Patients with single-station N2 (n=8/86) experienced pCR benefit with perioperative chemoimmunotherapy versus neoadjuvant chemotherapy alone (unweighted difference: 42.1% [95% CI: 19.9%–64.3%]), whereas benefit appeared to be lower in multi-station disease (unweighted difference: 26.4% [95% CI: −1%, 53.7%]) (78). In the Phase III CheckMate 77T trial, patients with N2 NSCLC appeared to experience EFS benefit with perioperative chemoimmunotherapy versus chemotherapy alone, regardless of whether this was single-station (HR: 0.50 [95% CI: 0.30–0.83]) or multi-station (HR: 0.48 [95% CI: 0.25–0.94]) disease (59). These EFS data were mirrored in the AEGEAN trial, but pCR benefit was less pronounced in patients with multi-station disease (difference in pCR rate: single-station N2, 13.9% [95% CI: 6.6–21.7]; multi-station N2, 3.8% [95% CI: −9.2, 18.8]) (76). Given the latest data, the proposed AJCC-UICC 9^th^ edition of TNM staging aims to reflect differences in clinical stage depending on whether patients are found to have single-station (N2a) or multi-station (N2b) disease across levels T1a–T3, which will be important to inform differences in management strategy (79).

It is important to note that the utility of clinical stage for determining optimal treatment approach is limited by the accuracy of upfront staging. A large, retrospective examination of patients with cT1cN0M0 NSCLC (categorized as clinical Stage I per AJCC-UICC 8^th^ edition) found that as high as 14.5% of patients who received lobectomy and 6.6% who received segmentectomy had pathologic nodal upstaging to pN1+ (Stage II+ per AJCC-UICC 8^th^ edition) (80). A UK-based retrospective evaluation found that one-third of patients with single-station N2 Stage III NSCLC by endobronchial ultrasound were upstaged to multi-station disease (81). These results were similar to those reported by a prior study in patients from the control group of randomized controlled trials of neoadjuvant chemotherapy in clinical Stage I–IIIA disease, which estimated that the discrepancies between clinical and pathologic T and N staging may have led to different treatment decisions in 10% and 38% of patients, respectively (82). Accurate, comprehensive staging is therefore critical to inform appropriate treatment strategies for all patients with NSCLC.

## 6 Response assessment in NSCLC and the potential for de-escalation/escalation of treatment

DFS and EFS are accepted surrogate endpoints in NSCLC, allowing an early evaluation of clinical benefit (83). Research is also focusing on providing actionable response assessments through alternative endpoints with more rapid readouts, enabling clinicians to escalate or de-escalate therapy in a personalized approach that could potentially improve outcomes while managing the risk of over- or undertreatment in resectable NSCLC (84).

### 6.1 Pathologic endpoints

Pathologic endpoints permit evaluation of response to therapy without the need for long time frames and are becoming routinely integrated into immunotherapy trials with a neoadjuvant component (5, 85). In a 2024 meta-analysis of randomized Phase III trials of chemoimmunotherapy versus chemotherapy alone in resectable NSCLC, there was a robust patient-level correlation observed between pCR and MPR and 2-year EFS (*R*^2^ = 0.82 and 0.81, respectively) (86). Notably, this study did not find correlation between pathologic assessment and 2-year OS (*R*^2^ = 0.55 for pCR and *R*^2^ = 0.52 for MPR). A second meta-analysis (also published in 2024) of neoadjuvant treatment (including targeted therapies, chemotherapy, and concomitant radiotherapy) found that OS was higher in patients who achieved pCR and MPR than in those who did not (HR: 0.49 [95% CI: 0.42–0.57] and HR: 0.36 [95% CI: 0.29–0.44], respectively) (87). Further data are needed to comprehensively validate pathologic response as an adequate surrogate for OS in immunotherapy trials; despite this, given the strong patient-level association observed, pathologic assessments yield valuable data to inform treatment decision-making by clinicians (86, 87).

In particular, pCR has been proposed as a response biomarker to guide the use of adjuvant immunotherapy after neoadjuvant chemoimmunotherapy and surgery, theoretically limiting exposure to potential treatment-related toxicities in patients who would not benefit from continued treatment (88, 89). At present, pCR assessment appears to show high reproducer reliability, with strong inter-rater agreement demonstrated between pulmonary pathologists when assessing slides from patients with resected NSCLC who have received neoadjuvant immunotherapy (inter-rater agreement: 0.94 [95% CI: 0.89–0.98]) (90). This supports the feasibility of pCR as a response biomarker; however, it is worth noting that it can be challenging to identify the tumor bed, and interobserver variability may occur (particularly between community pathologists) (91). Additionally, pCR is not a perfect biomarker, as a significant proportion of patients who achieve pCR will still experience disease recurrence (91). Finally, based on exploratory analyses from KEYNOTE-671 and CheckMate 77T, patients appeared to experience EFS benefit from perioperative immunotherapy plus neoadjuvant chemotherapy regardless of pCR status, relative to neoadjuvant chemotherapy alone (58, 62). This was mirrored by an exploratory, indirect patient-matched analysis from the CheckMate 816 versus CheckMate 77T trials, which found that patients appeared to experience EFS benefit from perioperative versus neoadjuvant immunotherapy regardless of pCR status (pCR: HR: 0.58; 95% CI: 0.14–2.40; non-pCR: HR: 0.65; 95% CI: 0.40–1.06) (92). In the 5-year analysis of CheckMate 816, exploratory analyses indicated that pCR status was prognostic for OS (pCR versus no pCR, HR: 0.11 [95%: 0.04–0.36]) (44). Taken together, the available data are currently insufficient to support utilization of pCR status to guide receipt of adjuvant immunotherapy following a neoadjuvant chemoimmunotherapy regimen, and clinicians should continue to make treatment decisions based on individual patient assessment (91). Although there are currently no Phase III trials designed to prospectively evaluate stratification of adjuvant treatment based on pCR status in NSCLC, a Phase III trial of adjuvant pembrolizumab versus observation in patients with breast cancer who have achieved pCR with neoadjuvant pembrolizumab plus chemotherapy is currently recruiting (as of the timing of publication of this review) and may yield some insights into this approach (93).

### 6.2 Liquid biopsy (MRD)

Liquid biopsy represents a minimally invasive method to detect biomarkers in fluids (e.g. blood and cerebrospinal fluid). Liquid biopsy allows detection of circulating tumor DNA (ctDNA) in patients with solid tumors, derived from cells that slough off the primary tumor/metastatic sites and enter the bloodstream (94). Tumor-informed assays involve using a tumor sample to inform the tracking of mutations in ctDNA and are associated with higher sensitivity than tumor-agnostic assays, which rely on a predefined panel of lung cancer–associated alterations for ctDNA tracking (95). Selecting between these approaches is primarily driven by tumor tissue availability (95). Detection of ctDNA by liquid biopsy represents an exciting tool to help determine the presence of MRD following curative-intent treatment in resectable NSCLC, facilitating response monitoring and risk stratification. Of particular relevance in the resectable setting, MRD monitoring presents the opportunity to tailor the adjuvant treatment strategy according to a patient’s response to neoadjuvant therapy.

A 2023 meta-analysis of ctDNA for MRD detection in lung cancer found that ctDNA represents a highly specific tool to detect disease recurrence, with ctDNA positivity following treatment associated with worse relapse-free survival (RFS; HR: 8.32) and OS (HR: 4.73) compared with ctDNA negativity (96). In resectable NSCLC specifically, post-treatment MRD positivity by landmark analysis correlated significantly with time to progression (*p*< 0.001), DFS (*p* < 0.001), EFS (*p* = 0.01), RFS (*p* < 0.001), and OS (*p*< 0.001), with strong prognostic value for both node-positive and node-negative patients (97, 98). Data from both CheckMate 77T and CheckMate 816 indicate that around half of patients who show ctDNA clearance do not achieve pCR, suggesting that ctDNA clearance may not represent a suitable prognostic factor for pathological outcomes following neoadjuvant chemoimmunotherapy (44, 59). However, data from CheckMate 816 suggest that failure to clear ctDNA may be associated with poorer 5-year OS, indicating its potential as a biomarker to guide future treatment intensification strategies (44).

In CheckMate 816, tumor-informed ctDNA negativity following three cycles of neoadjuvant nivolumab plus chemotherapy numerically correlated with higher rates of EFS and pCR, although this was not statistically evaluated (5). Furthermore, at the 5-year analysis, exploratory ctDNA analyses suggested that ctDNA clearance was associated with longer OS (ctDNA clearance versus no ctDNA clearance, HR: 0.38 [95% CI: 0.15–1.00); however, small patient numbers limit interpretation of these data (44). The AEGEAN trial found that earlier ctDNA negativity was associated with a higher likelihood of pCR (durvalumab: 50.0% versus 15.1%; placebo: 14.3% versus 3.1%) and MPR (durvalumab: 66.7% versus 35.8%; placebo: 38.1% versus 12.5%) (99). In the adjuvant setting, postoperative and post-chemotherapy tumor-informed ctDNA positivity in IMpower010 was found to be a negative prognostic factor for DFS, over 5 years of follow-up (100). However, MRD has been found to have poor sensitivity (pooled sensitivity: landmark, 0.41 [95% CI: 0.35–0.46]; surveillance, 0.76 [95% CI: 0.70–0.82]), indicating that negative ctDNA alone is not sufficient to indicate the absence of relapse in all patients (96). In an exploratory biomarker analysis of CheckMate 77T, achieving both ctDNA clearance and pCR appeared to be associated with higher EFS than either factor alone for patients treated with perioperative nivolumab plus chemotherapy (59). These data suggest that the use of the two response assessments together may be suitable as a prognostic factor for EFS and may represent an interesting avenue for further research.

Qiu B, et al. proposed that ctDNA positivity could be used as a prognostic marker to guide adjuvant chemotherapy in Stage II–III resected NSCLC, finding that patients who were ctDNA-positive following surgery experienced RFS benefit from adjuvant chemotherapy (*p* < 0.05), whereas patients who were ctDNA-negative had a low risk of relapse regardless of whether or not chemotherapy was administered (*p* = 0.46) (101). They found that a combinatorial approach (a so-called ‘joint model’) encompassing ctDNA, radiologic imaging, and clinical monitoring outperformed the traditional Cox models in predicting recurrence status at both 12 and 15 months following surgery. A combination of ctDNA and radiological tumor volume analysis has also been proposed to improve the low sensitivity of ctDNA analysis alone; this combination was found to accurately stratify patients as high versus low risk of relapse and survival in early-stage NSCLC (102). In the future, multifactorial approaches such as these may facilitate more timely therapeutic intervention and personalized treatment approaches in resectable NSCLC; however, ctDNA utility is currently restricted to prognostication only, as it is not yet a sensitive enough biomarker to guide treatment decisions.

## 7 Selecting between treatment approaches in resectable NSCLC

Both neoadjuvant and adjuvant chemoimmunotherapy approaches have demonstrated the potential to cure some patients without the need for a perioperative approach (5, 22, 24). However, to date, there are no head-to-head trials comparing these different treatment approaches, nor have sufficiently robust predictors of response been established to identify patients likely to be cured.

Forde PM, et al. conducted a patient-level data analysis of CheckMate 77T and CheckMate 816 to provide an indirect comparison between neoadjuvant nivolumab and perioperative nivolumab (92). Eligible patients received neoadjuvant nivolumab plus chemotherapy followed by definitive surgery and ≥1 dose of adjuvant nivolumab in CheckMate 77T, or neoadjuvant nivolumab plus chemotherapy as part of the CheckMate 816 trial. Perioperative nivolumab was found to be associated with improved EFS compared with neoadjuvant nivolumab (HR: 0.61 [95% CI: 0.39–0.97]). In addition, the subgroup of patients with PD-L1 <1% appeared to benefit from perioperative immunotherapy (EFS HR: 0.51 [95% CI: 0.28–0.93]), whereas data in patients with PD-L1 ≥1% were suggestive of no discernible difference (EFS HR: 0.86 [95% CI: 0.44–1.70]). Perioperative versus neoadjuvant nivolumab was associated with a numerical improvement in EFS, regardless of tumor stage (staging was aligned between the 7^th^ and 8^th^ AJCC-UICC criteria used by the CheckMate 77T and CheckMate 816 studies, respectively; Stage IB–II HR: 0.53 [95% CI: 0.25–1.11]; Stage III HR: 0.63 [95% CI: 0.37–1.07]). Finally, patients with pCR showed a risk reduction for EFS with perioperative versus neoadjuvant nivolumab, compared with those who did not achieve pCR (see *Response assessment in NSCLC and the potential for de-escalation/escalation of treatment*) (92). Patients in both trials showed similar rates of all-cause adverse events (AEs), with higher rates of AEs leading to discontinuation, treatment-related AEs, and serious AEs with perioperative nivolumab, although a slightly higher rate of surgery-related AEs (defined as AEs reported within 90 days of definitive surgery) was experienced with neoadjuvant nivolumab (92).

As the gold-standard endpoint in NSCLC, landmark OS data from neoadjuvant and perioperative trials may help to inform clinical decision-making. CheckMate 816 reported 5-year OS rates of 65.4% with neoadjuvant nivolumab plus chemotherapy versus 55.0% with chemotherapy alone, and a significant OS improvement (43, 44). To date, KEYNOTE-671 represents the only perioperative trial with a comparable duration of follow-up, reporting 4-year OS rates of 68.0% with perioperative pembrolizumab versus 56.7% with chemotherapy alone. Although the validity of cross-trial comparisons is limited, a clearer picture of the optimal treatment approach in resectable NSCLC may begin to emerge as data from more trials mature.

Notably, the FDA has determined that the traditional two-arm design of current perioperative trials does not allow for within-trial assessment of the relative contribution of neoadjuvant versus adjuvant phases of treatment, without which it remains challenging to establish the potential for overtreatment (103). Furthermore, as approaches evolve and add-on therapies are considered for an immunotherapy backbone in the resectable stage, the optimal duration and timing of therapy may become less and less clear (103). As a result, the FDA has called for use of alternative trial designs, including multi-arm trials or trials that incorporate re-randomization of patients following surgery, to allow direct comparison of treatment effect in the neoadjuvant versus adjuvant portion of the trial (103). The planned open-label, randomized, Phase III ADOPT-lung trial is designed to determine whether additional adjuvant treatment with durvalumab following neoadjuvant durvalumab plus chemotherapy yields a DFS benefit, and the results will be important for guiding the optimal treatment approach in this setting (104). Clinicians should continue to assess risk–benefit on an individual patient basis to guide treatment decisions in the curative-intent setting, based on the robust, long-term benefit conferred with the appropriate utilization of immunotherapy in the resectable setting.

## 8 Conclusions

There is clear rationale for the use of immunotherapy treatment for resectable NSCLC; however, the optimal timing of treatment (adjuvant, neoadjuvant, or perioperative) remains to be determined. The current evidence is of limited maturity, with inconsistencies in patient outcomes between clinical trials and a lack of head-to-head data between different immunotherapy regimens. As a result, there is a lack of alignment between current treatment guidelines (e.g. IASLC and NCCN), and updates to recommendations from other organizations are pending (e.g. ASCO and the European Society for Medical Oncology), leaving clinicians to interpret data based on trends observed between clinical trials (6, 7, 11, 21, 105).

Several factors may inform the optimal use of immunotherapy. Firstly, as a key biomarker, PD-L1 expression level appears to have prognostic utility in the neoadjuvant setting; however, patients appear to experience benefit from neoadjuvant immunotherapy regardless of PD-L1 status (5, 62, 71, 106). Patients with higher PD-L1 expression appear to experience greater benefit from chemoimmunotherapy in the perioperative setting; however, results differ between regimens (54, 56, 58, 60). In the adjuvant setting, there remains insufficient evidence to recommend immunotherapy in patients with PD-L1-negative tumors (22, 24). Secondly, when considering the role of clinical stage in patient selection, patients with Stage III NSCLC appear to experience the most benefit from immunotherapy-containing regimens (70, 73). In patients with Stage II NSCLC, meta-analyses indicate that neoadjuvant immunotherapy is beneficial, although the only trials to show clear benefit with a neoadjuvant-containing regimen to date are the perioperative trials KEYNOTE-671 and RATIONALE-315 (5, 56, 58, 60–62). Furthermore, evolving data on the influence of N2 involvement may provide nuance to the determination of optimal immunotherapy timing by clinical stage. Presently, data in patients with N2 disease are restricted to the perioperative setting and indicate that, although EFS benefit may be observed regardless of nodal stage, pCR benefit with perioperative chemoimmunotherapy may be greatest in patients with single-station involvement (56, 78). Future studies, including those that stage patients according to the more granular AJCC-UICC 9^th^ edition of TNM staging, will be important to inform on the potential benefit of immunotherapy by clinical and nodal stage.

Identifying reliable response biomarkers is similarly important. Although not yet validated as a surrogate survival endpoint in this setting, pCR is widely recognized as a useful measure to assess short-term treatment outcomes with neoadjuvant immunotherapy (86, 87). In the future, pCR may aid the identification of patients who could be spared subsequent adjuvant immunotherapy (88). Assessment of MRD via liquid biopsy represents a highly specific mechanism to identify the presence of tumor cells following neoadjuvant treatment and surgery; however, it currently lacks sufficient sensitivity as a standalone tool to guide treatment decision-making in resectable NSCLC (53, 96). With further study and refinement, MRD may be best used alongside clinical and radiologic outcomes to guide the optimal treatment approach (102).

Given the current challenges facing clinicians in the determination of optimal treatment strategy, multidisciplinary team assessment is recommended to guide the application of immunotherapy for patients with resectable NSCLC (6, 7). The close interplay between neoadjuvant treatment and resectability, as well as between pathologic responses and patient outcomes in the adjuvant setting, emphasizes the need for interdisciplinary alignment on the timing of immunotherapy (20, 21). As prognostic and response biomarkers continue to evolve, personalization of treatment approaches becomes an ever more realistic goal; as such, it is essential that multidisciplinary working practices are established and refined to optimize immunotherapy treatment and drive improved patient outcomes in resectable NSCLC.
