# Correction: Unlocking the potential of immunotherapy for patients with resectable non–small cell lung cancer

**DOI:** 10.3389/fonc.2026.1988694

**Published:** 2026-09-25

**Authors:** Sandip Pravin Patel, Justin Gainor, Steven Kao, Se-Hoon Lee, Tony Mok, Riyaz Shah, Caicun Zhou, Solange Peters

**Affiliations:** 1 UC San Diego Moores Cancer Center, San Diego, CA, United States; 2 Massachusetts General Hospital, Boston, MA, United States; 3 Chris O’Brien Lifehouse, Camperdown, NSW, Australia; 4 Samsung Medical Center, Sungkyunkwan University School of Medicine, Seoul, Republic of Korea; 5 State Key Laboratory of Translational Oncology, Department of Clinical Oncology, TheChinese University of Hong Kong, Hong Kong, Hong Kong SAR, China; 6 Maidstone Hospital, Maidstone, Kent, United Kingdom; 7 Shanghai East Hospital, Shanghai, China; 8 Lausanne University Hospital, Lausanne, Switzerland

**Keywords:** immunotherapy, resectable, NSCLC, locally advanced, neoadjuvant, perioperative, adjuvant, PD-L1

There was a mistake in **Figure 1** as published. The approval associated with the IMpower010 trial was incorrectly labelled as nivolumab rather than atezolizumab. The corrected **Figure 1** appears below.

**Figure 1:**
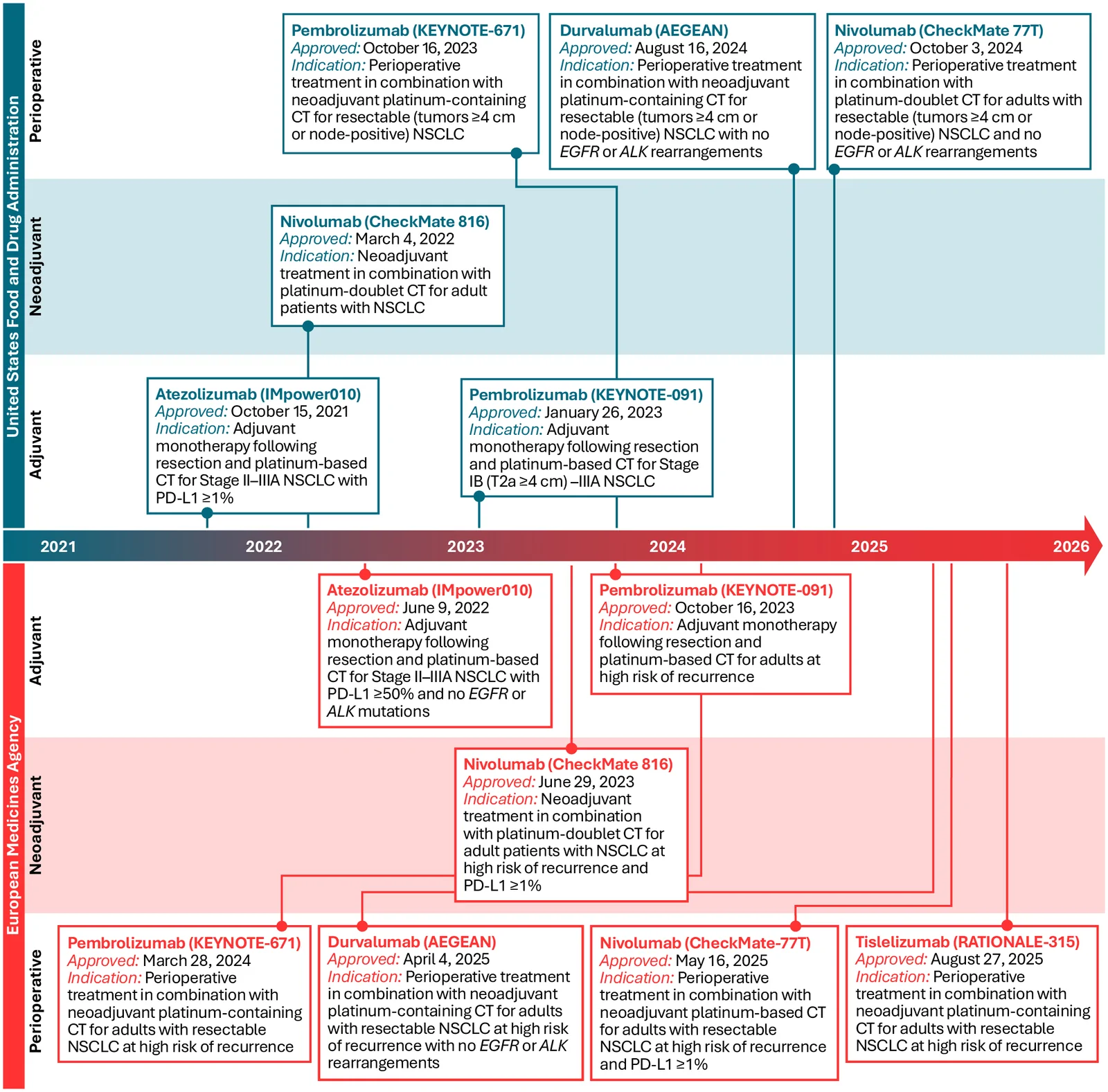
Timeline of key approvals of immunotherapy for the treatment of resectable NSCLC. CheckMate 816, IMpower010, and PEARLS/KEYNOTE-091 approvals followed enrollment based on the American Joint Committee on Cancer and the Union for International Cancer Control 7th edition. AEGEAN, CheckMate 77T, and KEYNOTE-671 approvals followed enrollment based on the American Joint Committee on Cancer and the Union for International Cancer Control 8th edition. CT, chemotherapy; NSCLC, non–small cell lung cancer; PD-L1, programmed-death ligand 1.

The original version of this article has been updated.

